# Investigation of carrier confinement in direct bandgap GeSn/SiGeSn 2D and 0D heterostructures

**DOI:** 10.1038/s41598-018-33820-1

**Published:** 2018-10-22

**Authors:** Denis Rainko, Zoran Ikonic, Nenad Vukmirović, Daniela Stange, Nils von den Driesch, Detlev Grützmacher, Dan Buca

**Affiliations:** 1 Peter Grünberg Institute (PGI 9) and JARA-Fundamentals of Future Information Technologies, Forschungszentrum Jülich, 52425 Germany; 2 Pollard Institute, School of Electronic and Electrical Engineering, University of Leeds, Leeds, United Kingdom; 3 Scientific Computing Laboratory, Center for the Study of Complex Systems, Institute of Physics Belgrade, University of Belgrade, 11080 Belgrade, Serbia

## Abstract

Since the first demonstration of lasing in direct bandgap GeSn semiconductors, the research efforts for the realization of electrically pumped group IV lasers monolithically integrated on Si have significantly intensified. This led to epitaxial studies of GeSn/SiGeSn hetero- and nanostructures, where charge carrier confinement strongly improves the radiative emission properties. Based on recent experimental literature data, in this report we discuss the advantages of GeSn/SiGeSn multi quantum well and quantum dot structures, aiming to propose a roadmap for group IV epitaxy. Calculations based on 8-band k∙p and effective mass method have been performed to determine band discontinuities, the energy difference between Γ- and L-valley conduction band edges, and optical properties such as material gain and optical cross section. The effects of these parameters are systematically analyzed for an experimentally achievable range of Sn (10 to 20 at.%) and Si (1 to 10 at.%) contents, as well as strain values (−1 to 1%). We show that charge carriers can be efficiently confined in the active region of optical devices for experimentally acceptable Sn contents in both multi quantum well and quantum dot configurations.

## Introduction

A significant outcome of technological development is the widespread use of network communications and data centers. The amount of information that are processed in these data centers is constantly increasing, reaching the limits of present day electronic chips concerning bandwidth and power consumption. Fundamental changes in chip design and data communication are therefore necessary to meet increasing demands^1^. Integration of photonic circuits with existing electronics is considered as one of these solutions, aiming to achieve on-chip or chip-to-chip communication via photons instead of electrons^2,3^.

State of the art chip technology uses Si-based materials, hence solutions based on group IV semiconductors are favored. Si, Ge and their alloys are indirect bandgap semiconductors, meaning that radiative processes in these materials are inefficient and slow. Therefore, finding suitable direct bandgap alloys based on group IV semiconductors is of primary importance.

Ge is an indirect bandgap semiconductor with the lowest indirect conduction band valley (at the L point) only 150 meV below the direct valley at the Γ point. Since Sn has a negative bandgap at the Γ point, partial replacement of Ge by Sn atoms changes the electronic band structure of GeSn so that the Γ-valley energy *E*_*Γ*_ decreases faster than the L-valley energy *E*_*L*_, increasing the directness *ΔE*_*L-Γ*_ = *E*_*L*_ *− E*_*Γ*_. Eventually, this results in a transition into a fundamental direct bandgap semiconductor at Sn concentrations around 8 at.% for unstrained GeSn^4,5^.

Breakthrough in epitaxial growth of GeSn alloys, mostly due to advances in low temperature chemical vapor deposition (CVD) methods and new precursors, enables alloy compositions that exceed the solubility limits of Sn in Ge by an order of magnitude^6–10^. These advances led to the successful demonstration of lasing from thick bulk GeSn/Ge/Si layers with Sn contents of 12.5 at.% at temperatures up to 90 K^5^. By incorporating even higher Sn concentrations, of up to 17.5 at.%, the lasing temperature limit reached 180 K^11,12^. On the way towards an electrically pumped room temperature laser, GeSn research benefits from the previous development of III-V semiconductor lasers, where heterostructures have been introduced in the 60 s^13^. To pursue this approach, a suitable barrier material for GeSn has to be found. Both the experiments and theoretical band alignment calculations indicate that Ge is unsuitable as a barrier material to confine carriers in direct bandgap GeSn wells^14^. On the other hand, a significant improvement in optical performance was demonstrated in GeSn/SiGeSn heterostructures^15,16^. Publications on GeSn/SiGeSn bulk heterostructures and quantum wells (QWs) agree on the suitability of SiGeSn as a barrier material for QWs, but an overview and investigation of the influence of doping and absorption processes on material gain is still missing^17,18^.

Regarding zero-dimensional GeSn/Si(Ge) quantum dots (QD), theoretical investigations indicate that no favorable band offset for electrons in the Γ-valley, *V*_*Γ*_ = *E*_*Γ*,*SiGeSn*_ − *E*_*Γ*,*GeSn*_, is possible if the direct bandgap (*ΔE*_*L-Γ*_ > 0 meV) in the dot is to be maintained^19^.

This work presents band energy calculations of GeSn/SiGeSn multi quantum wells (MQWs) and QDs for optoelectronic devices using 8-band k·p and effective mass methods. The range of Sn and Si contents has been chosen to cover both the epitaxial accessibility and the reliability of the element dependent bandgap interpolation procedure for alloys. The process of finding optimal parameters for efficient charge carrier confinement is described in detail. Furthermore, the results are analyzed for optical properties like material gain in doped and undoped structures, as well as optical cross section. The aim is to offer a solution for GeSn/SiGeSn heterostructures with the best chances to realize a room temperature group IV laser.

## Calculation Details

**Figure 1:**
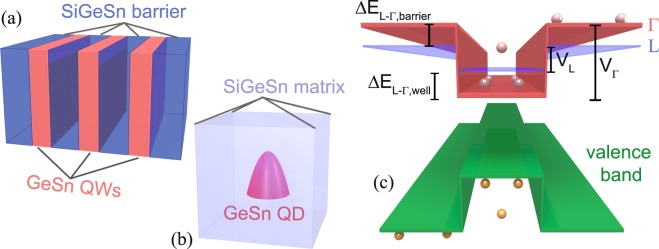
(**a**) Schematic layer structure of the 3 × {30 nm GeSn/40 nm SiGeSn} MQW and (**b**) a cone shaped GeSn QD embedded in a SiGeSn matrix as discussed in this work. (**c**) Type-I band alignment between well and barrier with the band discontinuities *V*_*Γ*_,_*L*_ = *E*_*Γ*,*L*,*SiGeSn*_ − *E*_*Γ*,*L*,*GeSn*_ and well directness *ΔE*_*L-Γ*_ denoted.

Particular MQW and QD heterostructures were representatively chosen to perform detailed calculations on the influence of strain and quantization using 8-band k·p method as it was formulated for the diamond/zinc blende lattice in ref.^20^. For the MQWs, the well thickness was chosen to be 30 nm, separated by 40 nm thick barriers as schematically shown in Fig. 1a. The reason for such high thicknesses is the negative influence on the directness of the well material. Thin wells cause a strong quantization, while a low barrier thickness introduces band splitting. Since the electron effective mass in GeSn is much lower for the Γ- than for the L-valley – and quantization energy reciprocally depends on the effective mass – decreasing the well/barrier thickness will decrease the energy difference between Γ and L. For GeSn well thicknesses below 10 nm quantization results in a transition back into an indirect semiconductor^14,21^. Due to rather thick barriers, and hence weak coupling of neighbouring wells (particularly for their ground states) the MQW calculations have been performed for structures with only three wells, which is good enough for investigating the material gain. Certainly, real MQW structures should have a larger number of wells in order to provide a good modal overlap with the active layer, and hence the modal gain not to be significantly lower than the material gain.

The crystal orientation in this work is taken to be (001), i.e. QWs are assumed to be grown on the (001) substrate surface, as it is typical in epitaxy^16^. The band structure calculation methods used in this work have been described elsewhere^19,20,22^. Bandgap energies ${E}^{{\rm{\Gamma }},L}$ of unstrained (Si)GeSn alloys have been calculated using the element concentrations (*x*_*Ge*_, *x*_*Sn*_, *x*_*Si*_) dependent interpolation, with bowing parameters ${b}^{{\rm{\Gamma }},L}$ derived from empirical pseudopotential method and experimentally from photoluminescence (PL) measurements:

1 $${E}^{{\rm{\Gamma }},L}={E}_{Ge}^{{\rm{\Gamma }},L}{x}_{Ge}+{E}_{si}^{{\rm{\Gamma }},L}{x}_{Si}+{E}_{Sn}^{{\rm{\Gamma }},L}{x}_{Sn}-{b}_{SiGe}^{{\rm{\Gamma }},L}{x}_{Ge}{x}_{Si}-{b}_{GeSn}^{{\rm{\Gamma }},L}{x}_{Ge}{x}_{Sn}-{b}_{SiSn}^{{\rm{\Gamma }},L}{x}_{Si}{x}_{Sn}$$

The temperature dependence of bandgaps of constituents was included via Varshni’s formula, and the effects of strain via deformation potentials. The values of the parameters used for 8-band k·p calculations are summarized in Table 1. A detailed description of band alignment calculations are added to the methods section.

**Table 1:** Parameters used for bulk bandgaps and 8-band k·p calculations.

Parameter	Si	Ge	Sn	SiGe	GeSn	SiSn
**Lattice constants and deformation potentials (ac: for conduct**. **band at Γ; av**, **bv & d: for valence band at Γ; a**_**L**_**: for conduct**. **band at L)**						
a_lattice_, b_lattice_ [Å]	5.4307^d^	5.6579^d^	6.4890^d^	0.026^h^	−0.041^g^	0
a_c_ [eV]	−10.06^d^	−8.24^j^	−6^d^			
a_v_ [eV]	2.46^j^	1.24^j^	1.58^k^			
b_v_ [eV]	−2.1^j^	−2.9^j^	−2.7^i^			
d [eV]	−4.8^a^	−5.3^a^	−4.1^m^			
a_L_ [eV]	−0.66^j^	−1.54^j^	−2.14^k^			
**Bandgaps**, **bandgap bowings and Varshni parameters (Δ**_**SO**_**: valence band spin-orbit splitting**, **d**_**i**_**: Temp**. **dependence of bowing parameters)**						
${{\rm{E}}}_{{\rm{bandgap}}}^{{\rm{\Gamma }}}$[eV]	4.185^a^	0.898^a^	−0.408^a^			
${{\rm{E}}}_{{\rm{bandgap}}}^{{\rm{L}}}$[eV]	2.176^i^	0.744^f^	0.1202^e^			
Δ_SO_ [eV]	0.044^n^	0.297°	0.80^p^			
b_Γ_ [eV] @ 0 K d_Γ_ [eV/K]				0.21^e^, 0	2.24^c^, −4·10^−4 c^	3.915^e^, 0
b_L_ [eV] @ 0 K, d_L_ [eV/K]				0.335^e^, 0	0.89^b^, −7·10^−4 b^	2.124^e^, 0
α_Varshni,Γ_ [eV/K] β_Γ_ [K]	3.91·10^−4f^, 125^f^	5.82·10^−4f^, 296^f^	0			
α_Varshni,L_ [eV/K] β_L_ [K]	4.774·10^−4f^, 235^f^	4.774·10^−4f^, 235^f^	0			
**Elastic constants**						
C_11_ [GPa]	165.77^a^	128.53^a^	69^a^			
C_12_ [GPa]	63.93^a^	48.26^a^	29.3^a^			
**Luttinger’s parameters of the 6-band model**						
γ_1_^L^	4.285^a^	13.38^a^	−12^k^			
γ_2_^L^	0.339^a^	4.24^a^	−8.45^k^			
γ_3_^L^	1.446^a^	5.69^a^	−6.84^k^			
**Optical energies**						
E_P_ [eV]	21.6^a^	26.3^a^	23.8^l^			

^a^ref.^43^, ^b^ref.^44^, ^c^ref.^45^, ^d^ref.^46^, ^e^ref.^47^, ^f^ref.^48^, ^g^ref.^9^, ^h^ref.^49^, ^i^ref.^50^, ^j^ref.^51^, ^k^ref.^52^, ^l^ref.^53^, ^m^ref.^54^, ^n^ref.^55^, ^o^ref.^56^, ^p^ref.^57^.

The electron and hole quantized states in QW structures were calculated by effective mass method with nonparabolicities (all these values, as well as band energies, coming from 8-band k·p model with strain included), using the finite-difference method^23^, which gives the same results for subband edges (at zero in-plane wave vector) as the full k·p calculation. L-valley energies have been calculated using the simple effective mass method, which is sufficient to get accurate energies of lowest states in the L-valley, while k·p calculations would require a higher number of bands to be considered^19^.

Similar as in quantum wells, to calculate the energies of QD electron and hole states originating from Γ-valley we use the 8-band k·p model with the effect of strain taken into account. We consider cone shaped dots with a cylindrical symmetry (Fig. 1b), which is a reasonable approximation to experimentally more common pyramidal shapes, and significantly reduces the computational effort. Moreover, since there are no experimental data on GeSn QDs, this assumption is an appropriate first step to gain insight into the band alignment in GeSn QDs. Thus, cone shaped structures were chosen with a base diameter of 20 nm and a height of 30 nm. Consequently, electron and hole states can be labeled by the quantum number *m*_*f*_, which represents the z-component of the total quasi angular momentum operator. The eigenvalue problem of the QD Hamiltonian was numerically solved using the orthonormal function expansion method^22^. Strain distribution was modeled using a continuum mechanical model and was numerically found using the finite element method^22^. To find the energies of QD conduction band states originating from the L-valley, we used the effective mass model including strain effects, which was also numerically solved using the orthonormal function expansion method, as described in ref.^22^.

The elemental concentrations considered here were chosen for wells/QDs to be 8/15 at.% < *x*_*Sn*_ < 20 at.%, and 8/10 at.% < *x*_*Sn*_ < 20 at.% and 1 at.% < *x*_*Si*_ < 10 at.% for the SiGeSn barrier/matrix to be in the experimentally achievable range in CVD experiments. The MQW barriers were initially considered biaxially strained and pseudomorphically grown on top of unstrained wells and substrate. The strain in the wells, related to strain-balance requirements, is accounted for later. In case of the QDs, the strain stems from dots embedded in a matrix of different composition, being unstrained far away from the dot.

Gain calculations for intrinsic and doped MQWs were performed, and free carrier absorption (FCA), calculated for bulk Ge_0.84_Sn_0.16_, was also included to find the net gain. Gain calculations are described in the methods section at the end, while FCA calculations use the second order perturbation model for bulk semiconductors described in ref.^24^. This model includes acoustic phonon scattering, deformation potential scattering (L-valley), intervalley scattering, ionized impurity scattering and alloy scattering.

Similarly as in MQW calculations, a specific QD structure was chosen to investigate the optical cross section ${\sigma }_{if}^{\varepsilon }$ (see method section) and the influence of biaxial strain and Si content in the barrier.

## Results and Discussion

### GeSn/SiGeSn MQW heterostructures

All considered heterostructures were analyzed for their carrier confinement, aiming at high conduction band discontinuities of Γ- and L-valleys between barrier and well: V_Γ_ ≥ 100 meV, V_L_ ≥ 0 meV (*V*_*L*_ = *E*_*L*,*SiGeSn*_ − *E*_*L*,*GeSn*_). A high band discontinuity for the Γ-valley is needed, as has been demonstrated in PL measurements on GeSn/SiGeSn MQWs in which presumably low band discontinuities lead, at higher temperatures, to no measurable benefits of heterostructures, compared to bulk structures [Paper accepted to be published in ACS Photonics]. Furthermore, the well region has to maintain its direct bandgap (*ΔE*_*L-Γ*,*well*_ ≥ 0 meV), while for the barriers an indirect bandgap (*ΔE*_*L-Γ*,*barrier*_ ≤ 0 meV) is considered preferable in this work. The latter may not be a very strict requirement. It can be argued that an indirect barrier material is less efficient in contributing to losses by radiative processes. Also, theoretical considerations for III-V heterostructures predict lower thermionic currents at interfaces between direct and indirect semiconductors^25^. However, it is presently uncertain whether the injected charge carriers remain long enough in a direct bandgap barrier to recombine radiatively, and whether the non-radiative losses in it are weaker than in an indirect bandgap barrier.

The directness of the GeSn well, *ΔE*_*L-Γ*,*well*_, generally increases with its Sn content. At the same time, when increasing the Si content inside the SiGeSn barrier, the band discontinuity between SiGeSn barriers and GeSn wells increases, an effect which can be observed in all calculated structures. The Si content range investigated in this work is limited to values of 10 at.%, which is realistic for SiGeSn layers grown by CVD. For MBE processes higher Si contents have been achieved^26^. Under the assumption of a constant bandgap bowing of SiSn at the Γ point (see discussion of b_SiSn,Γ_ in method section), it can be expected that Si contents sligthly above 10 at.% will lead to a stronger carrier confinement. But since it is unknown what effects might occur for high Si contents, this has to be investigated experimentally.

**Figure 2:**
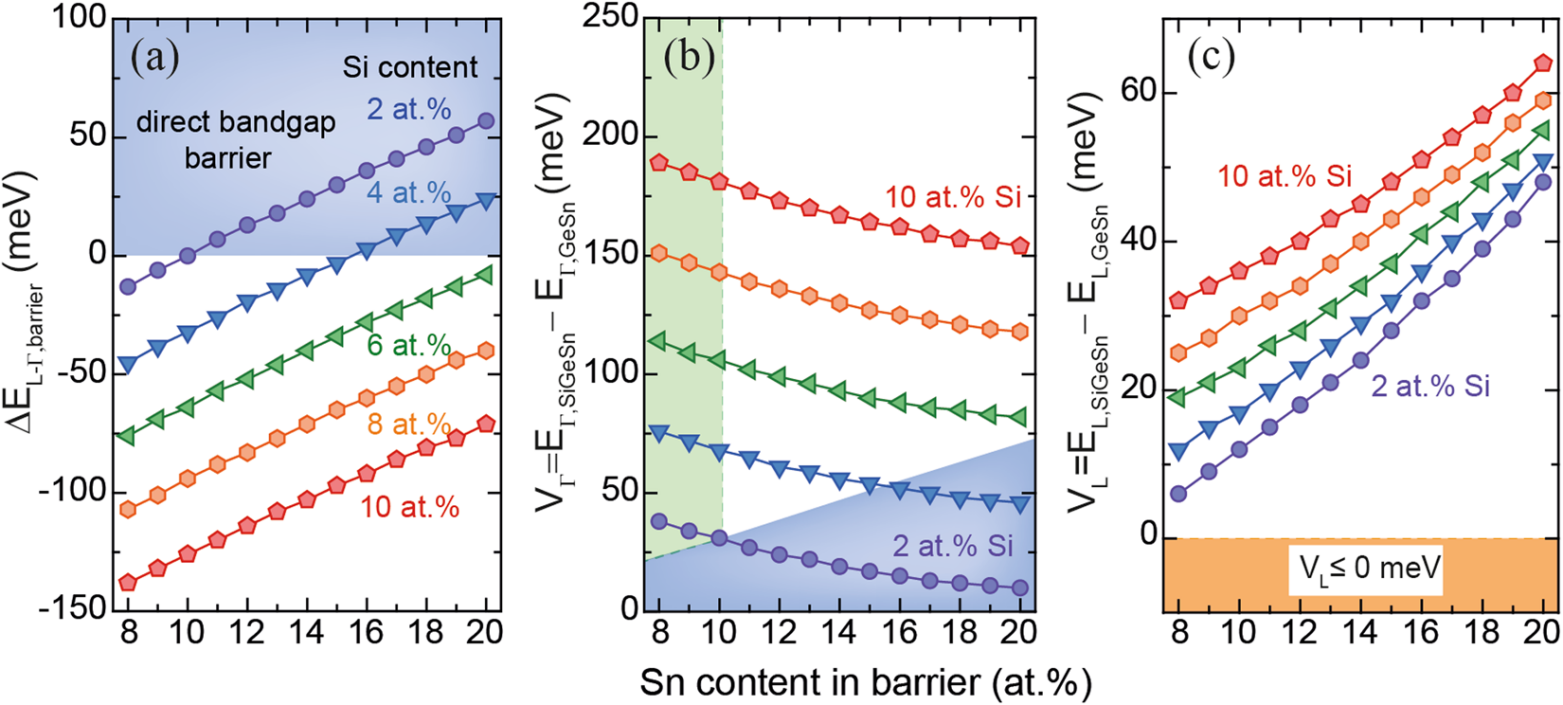
Directness of SiGeSn barrier (**a**), band discontinuity for Γ- (**b**) and L-valleys (**c**) for a Ge_0.92_Sn_0.08_/SiGeSn MQW for different Sn contents in the barrier. The colored areas indicate regions where SiGeSn is an indirect semiconductor (blue), band discontinuities for L are negative (orange), and which correspond to CVD realistic layers (green).

To illustrate the process of finding the strongest carrier confinement in e.g. Ge_0.92_Sn_0.08_/SiGeSn MQWs, the directness of SiGeSn, *ΔE*_*L-Γ*,*barrier*_, (Fig. 2a) and the band discontinuities for L- and Γ-valleys (Fig. 2b,c) are plotted for several barrier compositions. Since it was initially assumed that wells are unstrained, Sn contents >8 at.% guarantee that the GeSn well has a direct bandgap^27^. After excluding the region with a direct bandgap barrier (blue area), negative band discontinuity of the L-valleys (orange area), and including Sn contents close to the Sn content in the well (green area), which represent CVD-realistic Sn contents, a range of heterostructures which fulfill the previously stated requirements remains. Band discontinuities of 180 meV for the Γ-valley can be achieved, together with a directness *ΔE*_*L-Γ*_ in the well region of 160 meV.

**Figure 3:**
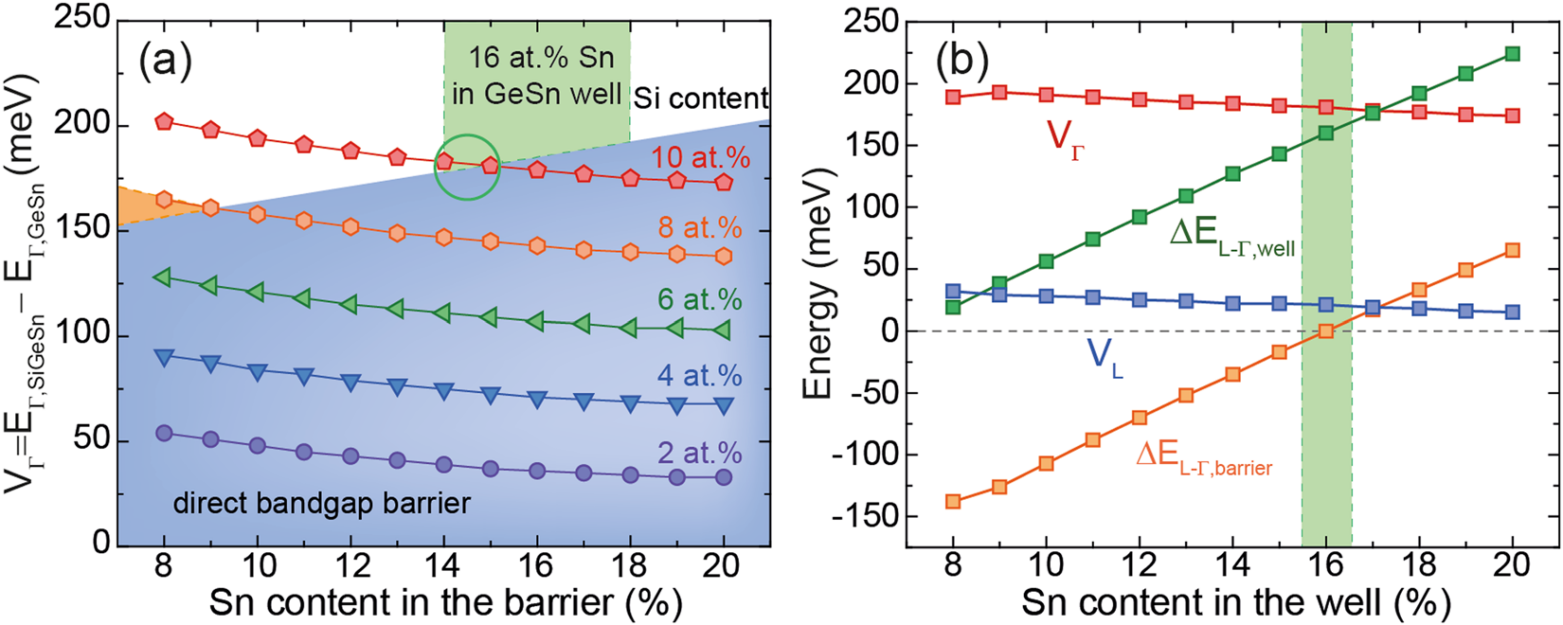
(**a**) Band discontinuities of the Γ-valley in a Ge_0.84_Sn_0.16_/SiGeSn MQW for different Sn contents in the barrier. (**b**) Directness of barrier and well, and band discontinuities for L- and Γ-valleys in dependence of the well Sn content in GeSn/SiGeSn MQWs achieving best band alignment for Γ.

On the other hand, when the Sn content in the well increases, e.g. from 8 at.% (Fig. 2b) to 16 at.% (Fig. 3a), the green area in Fig. 3a decreases, leaving only a small parameter space for good carrier confinement (marked by a green circle). The reason for this is solely the increase of *ΔE*_*L-Γ*_ in the SiGeSn barrier material, meaning that at high Sn contents the barrier becomes a direct bandgap semiconductor. Therefore, under the target requirements on carrier confinement, used in this work, high Sn contents in the barrier are not favorable, which has to be considered in the course of finding optimized laser structures. The issue of having a direct bandgap barrier can be overcome by increasing the compressive strain in the MQW or by higher Si contents, but the latter option is not presently realistic due to experimental limitations of CVD epitaxy. At an Sn content of 16 at.% in the well, the barrier (with a Si/Sn content of 10/16 at.%) becomes direct, which, as discussed above, is unfavorable and leaves only a small parameter space in the green marked areas (Fig. 3a). The material parameters in the green regions offer good carrier confinement, and are further filtered by choosing GeSn/SiGeSn stoichiometries with maximal band discontinuity at Γ. Extending the above procedure to a larger range of Sn contents in the well, leads to the results presented in Fig. 3b. For heavy holes (HH) and light holes (LH) the band discontinuities are found to be around 104 meV and 40 meV in all structures. The optimal Si and Sn contents in the barrier are 10 at.% Si and 1 at.% less Sn than in the well.

**Figure 4:**
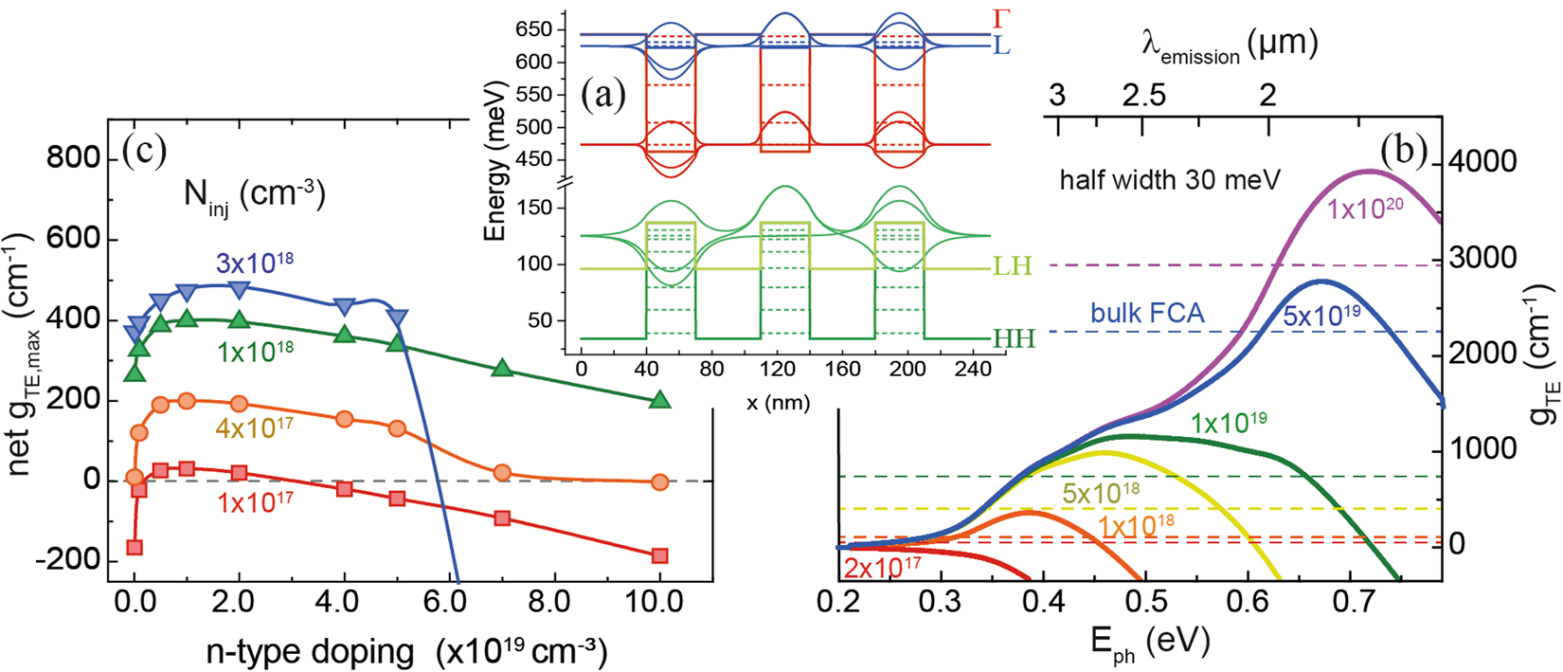
(**a**) Band diagram of an unstrained 3 × {30 nm Ge_0.84_Sn_0.16_/40 nm Si_0.10_Ge_0.75_Sn_0.15_} MQW. Dashed lines represent quantized energies and wavefunctions of ground states of each valley. (**b**) TE-mode material interband gain at 300 K for different injected carrier densities. Dashed lines indicate FCA values for bulk Ge_0.84_Sn_0.16_. (**c**) Net gain dependence on n-doping density, where FCA has been subtracted from interband gain.

Based on the results presented in Fig. 3, a 3 × {30 nm Ge_0.84_Sn_0.16_/ 40 nm Si_0.10_Ge_0.75_Sn_0.15_} MQW was chosen to calculate the material gain and the influence of strain and Si content on charge carrier confinement. This structure was chosen because of a trade-off between high band discontinuities and directness in the well, while maintaining an indirect barrier. The band structure of this specific MQW is shown in Fig. 4a. The material interband gain for different carrier injection densities *N*_*inj*_ at 300 K is given in Fig. 4b. The gain calculation assumes Lorentzian broadening with a half width of 30 meV at 300 K. Dashed lines indicate FCA values of bulk Ge_0.84_Sn_0.16_ to be deducted from this gain. Auger recombination has no direct influence on gain calculated as a function of carrier densities, though it can have a strong indirect influence, by decreasing the carrier lifetime and hence affecting the actual carrier densities achievable for a particular injection current or optical pump intensity. Since this paper gives the gain dependence on carrier density only, Auger recombination is not included in calculations. The injected carrier densities necessary for pure interband gain to overcome FCA losses are extracted to be of around 2 × 10^17^ cm^−3^. At carrier densities between 1 × 10^19^ cm^−3^ and 1 × 10^20^ cm^−3^ (purple graph in Fig. 4b) net gain reaches a minimum before increasing again at carrier intensities exceeding 1 × 10^20^ cm^−3^.

By doping the active well, the population of charge carriers in the Γ-valley can be increased, which can enhance the performance of optoelectronic devices. Our results show that including n-type doping into the active material has dramatic consequences on the material net gain (Fig. 4c). Since the FCA increases with doping, especially due to ionized impurity scattering, doping concentrations above 1 × 10^20^ cm^−3^ at moderate injection carrier densities of 1 × 10^18^ cm^−3^ (green graph in Fig. 4b) lead to a significant material gain with a maximum of 400 cm^−1^. For injection carrier concentrations above 3 × 10^18^ cm^−3^ (blue curve in Fig. 4b) the net material gain decreases drastically for low doping concentrations. In conclusion, only a limited range of doping concentrations is beneficial for the gain, while for higher doping the above mentioned limits cause a deterioration of the optical performance. This is in contrast to indirect bandgap Ge, where a strong doping is necessary to achieve any significant Γ population at all, but in direct bandgap GeSn too large n-type doping leads to a decrease of gain due to FCA.

**Figure 5:**
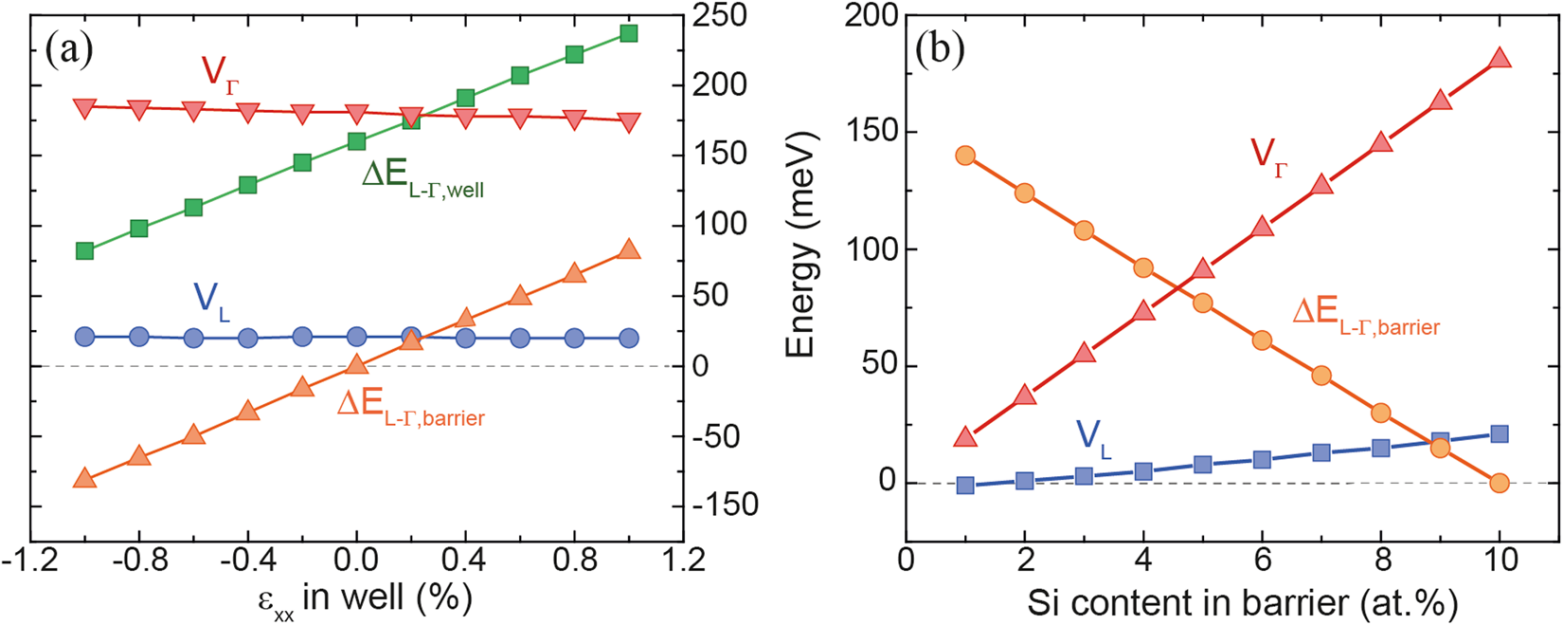
Influence of strain (**a**) and Si content in SiGeSn (**b**) on band alignment parameters for a Ge_0.84_Sn_0.16_/Si_0.10_Ge_0.75_Sn_0.15_ MQW.

From the epitaxial point of view, especially regarding CVD technology, it is realistic to grow compressively strained GeSn wells (*ε*_*xx*_ ≈ −0.5%) with low Si contents in the SiGeSn barriers (Fig. 5). On the other hand, tensile strain can be induced by strain engineering techniques like Si_3_N_4_ stressor layers, under-etching of mesa structures^28–31^ or even by hetero-epitaxy^31^. Therefore, we have also analyzed the influence of strain on band alignment for a range of strain values from −1% to 1% in the Ge_0.84_Sn_0.16_/Si_0.10_Ge_0.75_Sn_0.15_ MQW structure (Fig. 5a). Strain hardly influences the band discontinuity of Γ- and L-valleys. The slightly compressive strain also decreases directness of both the well and the barrier. However, while the well bandgap remains direct within the whole range of strain values, the bandgap of the barrier changes from direct to indirect as soon as the strain becomes compressive. (Fig. 5a).

With lowering the Si content in the barrier, the deteriorating effect on carrier confinement is significant. The value of *ΔE*_*L-Γ*_ in the barrier strongly increases, causing a transition into a direct bandgap barrier, and the band alignment also becomes worse (Fig. 5b).

**Figure 6:**
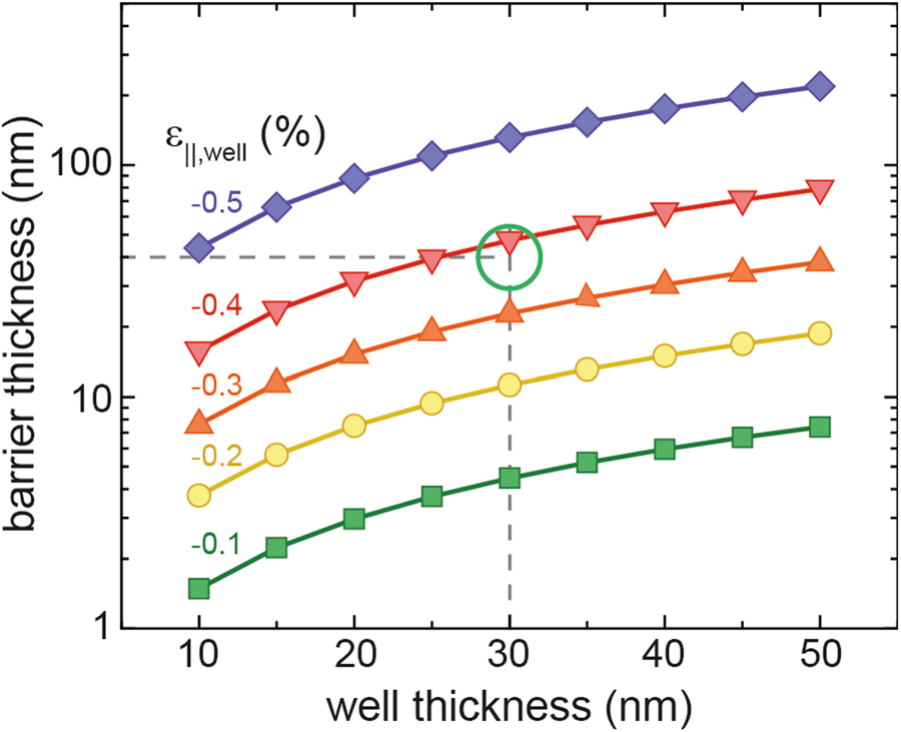
Strain balanced barrier and well thicknesses averaged for GeSn_0.08–0.20_/Si_0.10_GeSn_0.07–0.19_ heterostructures with *x*_*Sn*,*well*_ = x_Sn,*barrier*_ + 1 at.% for different strain values in the well.

In addition to the above discussed influence of strain, strain balance requirements were added to MQW calculations. From the experimental point of view, when designing MQW heterostructures, strain balanced structures are desirable in order to avoid plastic relaxation, which induces defects in the active region^6^. Therefore, strain balance calculations for several compressive strain values in the well have been performed according to ref.^32^ to determine the required barrier thickness to compensate the strain in the well. The Sn contents of well and barrier were chosen so that *x*_*Sn*,*well*_ = *x*_*Sn*,*barrier*_ + 1 at.%. The results show no significant dependence of barrier and well thickness on Sn content for a fixed strain value under the above described material parameter conditions. The deviation of the calculated barrier thickness is 2 nm for 45 nm thick barriers and 10 nm for 220 nm thick barriers. When increasing the well thickness, the required barrier thickness continuously increases, going from 1.5 nm for 10 nm thick wells and strain in the well of −0.1% (green line in Fig. 6) to around 7 nm for well thicknesses of 50 nm. When the compressive strain in the well is about −0.5%, barrier thicknesses of 220 nm are needed to strain balance 50 nm thick wells (purple line in Fig. 6). The dashed lines in Fig. 6 indicate the well and barrier thickness for the previously discussed structure. With a strain value of −0.4% inside the wells, the Ge_0.84_Sn_0.16_/Si_0.10_Ge_0.75_Sn_0.15_ MQW would be strain balanced, while still maintaining a good carrier confinement (Fig. 5a).

### QD calculations

**Figure 7:**
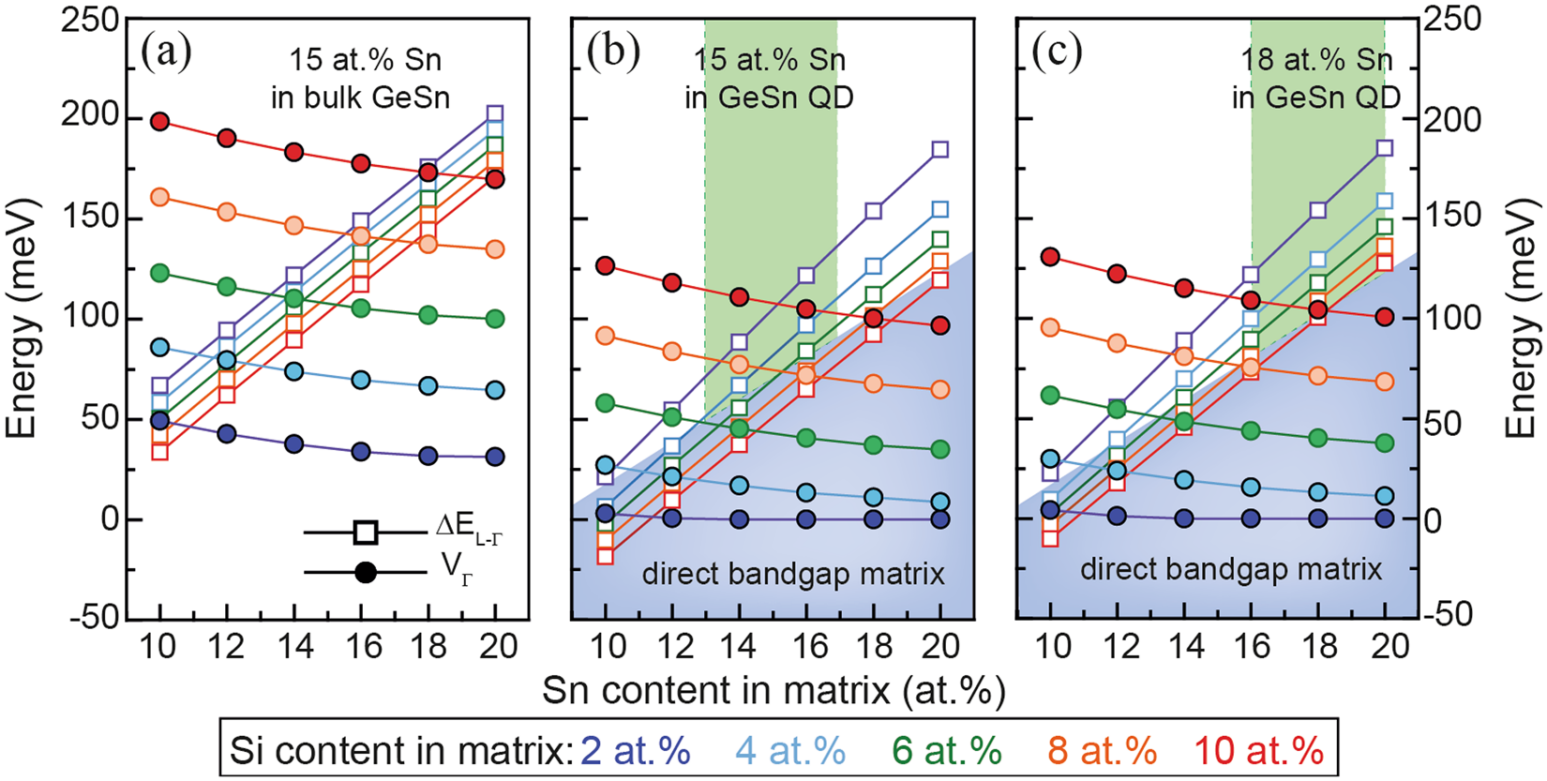
Directness of the potential well (empty squares) and band discontinuity at Γ (solid circles) for (**a**) bulk Ge_0.85_Sn_0.15_/SiGeSn, (**b**) a Ge_0.85_Sn_0.15_/SiGeSn QD and (**c**) Ge_0.82_Sn_0.18_/SiGeSn QD. The meaning of the colored areas is the same as described for MQW calculations.

The most important aspect of GeSn QDs is an even stronger influence of quantization in three dimensions on band alignment and directness, compared to the case of MQWs. Comparing the bulk (Fig. 7a) and QD results (Fig. 7b) for the band discontinuity *V*_*Γ*_, and *ΔE*_*L-Γ*_ inside the potential well for a Ge_0.85_Sn_0.15_ QD gives an average decrease of 75 meV in *V*_*Γ*_ and 50 meV decrease in *ΔE*_*L-Γ*_ when introducing quantization in three dimensions. Similar to the MQW calculations described above, a range of Si/Sn contents of the surrounding SiGeSn matrix was investigated. The same kind of general behavior for *V*_*Γ*_ and *ΔE*_*L-Γ*_ is observed here as was found for MQWs.

When applying the same process of finding optimal material parameters, as previously described for MQWs, structures with good carrier confinement are found, as indicated by the green area in Fig. 7. However, it is still unknown whether the Si/Sn contents for QD systems are limited similarly as in the case of bulk and QW heterostructures, since there are no publications on the epitaxial growth of GeSn QDs in a SiGeSn matrix.

It should be noted that for the QD calculations presented here the strain of the QD was completely determined by the lattice mismatch between the GeSn dot and the SiGeSn surrounding matrix, which is unstrained far away from the dot. This explains why there are nearly no differences in band discontinuity *V*_*Γ*_ and *ΔE*_*L-Γ*_ for QDs with different Sn contents (Fig. 7a,b). When increasing *x*_*Sn*,*dot*_ (for the same Si/Sn content in the matrix) – and therefore increasing *ΔE*_*L-Γ*,*dot*_ and *V*_*Γ*_ – the strain in the QD increases, which has the opposite effect of increasing the Sn content in the GeSn dot. For conical, hydrostatically strained QDs, these opposing effects are more pronounced than for MQW structures, and they largely cancel out each other, as can be seen in Fig. 7b,c.

**Figure 8:**
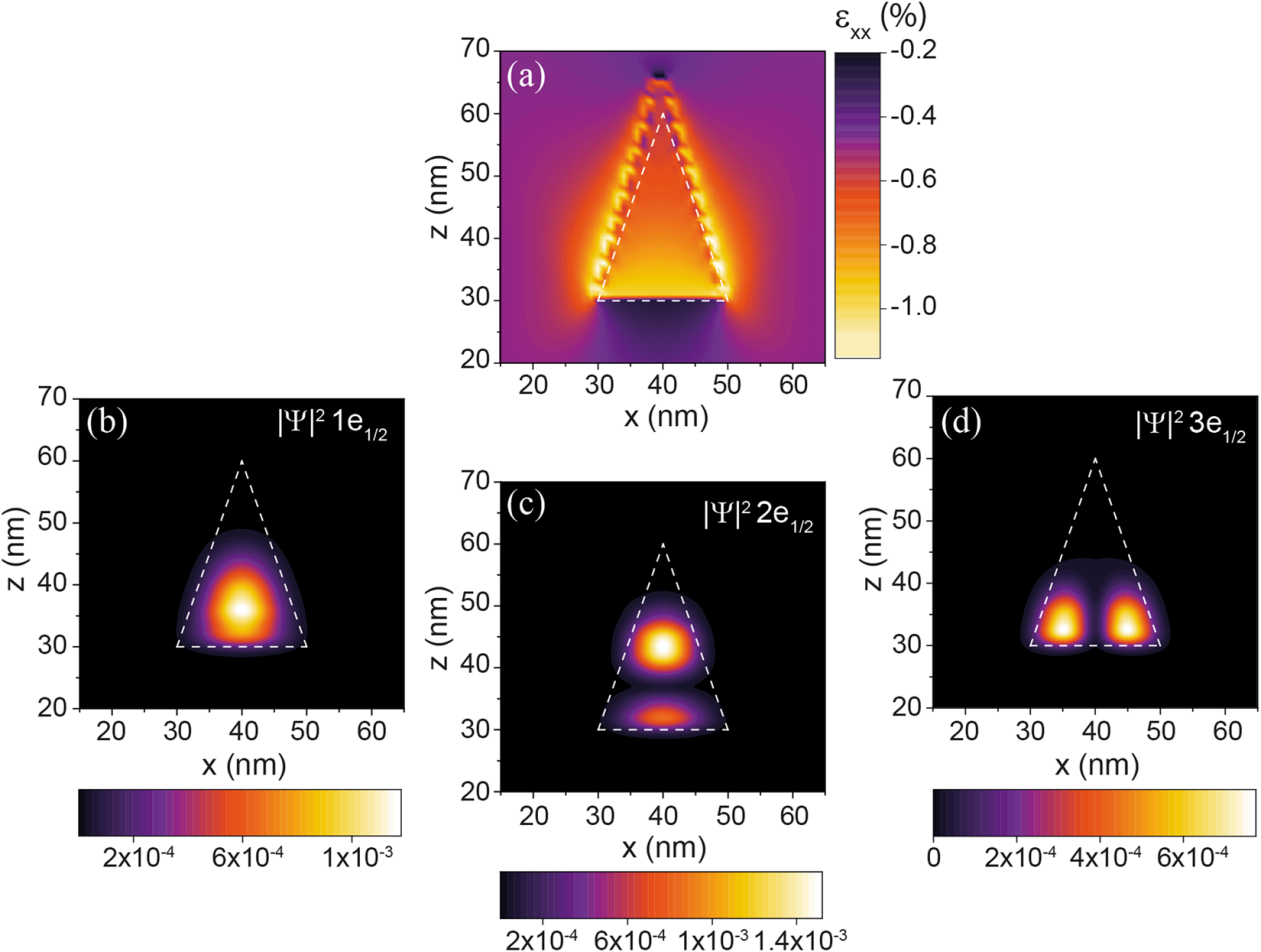
Strain field in the xz-plane of a biaxially strained Ge_0.82_Sn_0.18_/Si_0.10_Ge_0.74_Sn_0.16_ QD (**a**) and the wave function probability densities for the first 3 Γ-electron states with total angular momentum of *m*_*f*_ = 1/2 (**b**–**d**). Dashed lines indicate the geometry of a conical QD. Biaxial strain is induced by a Ge_0.90_Sn_0.10_ substrate.

There are many publications on the epitaxy of conical or lens shaped QDs in other material systems. In those, however, strain inside the matrix is usually biaxial and determined by the substrate^33,34^. To investigate more common cases from the epitaxy point of view, a Ge_0.82_Sn_0.18_ QD with a Si_0.10_Ge_0.74_Sn_0.16_ matrix was chosen, where biaxial strain was taken to exist in the matrix material. Strain was induced by GeSn substrates with Sn contents ranging from 10*–*20 at.%. The strain distribution in a Ge_0.82_Sn_0.18_ QD inside a Si_0.10_Ge_0.74_Sn_0.16_ matrix, itself biaxially strained by a Ge_0.90_Sn_0.10_ substrate, can be seen in Fig. 8a. Far away from the dot, the matrix adopts the strain value imposed by the substrate. As expected, compressive strain at the interfaces between dot and matrix exceeds relatively high values of 1%.

To investigate the carrier confinement in the above described QD, the wavefunction probability density *|Ψ|*^2^ was calculated for the first three Γ-electron states with angular momentum of *m*_*f*_ = 1/2 and plotted in the xz-plane (Fig. 8b–d). For all investigated QD structures, the first 6 Γ- electron states are confined in the dot, illustrating a strong localization of the electron wave function.

**Figure 9:**
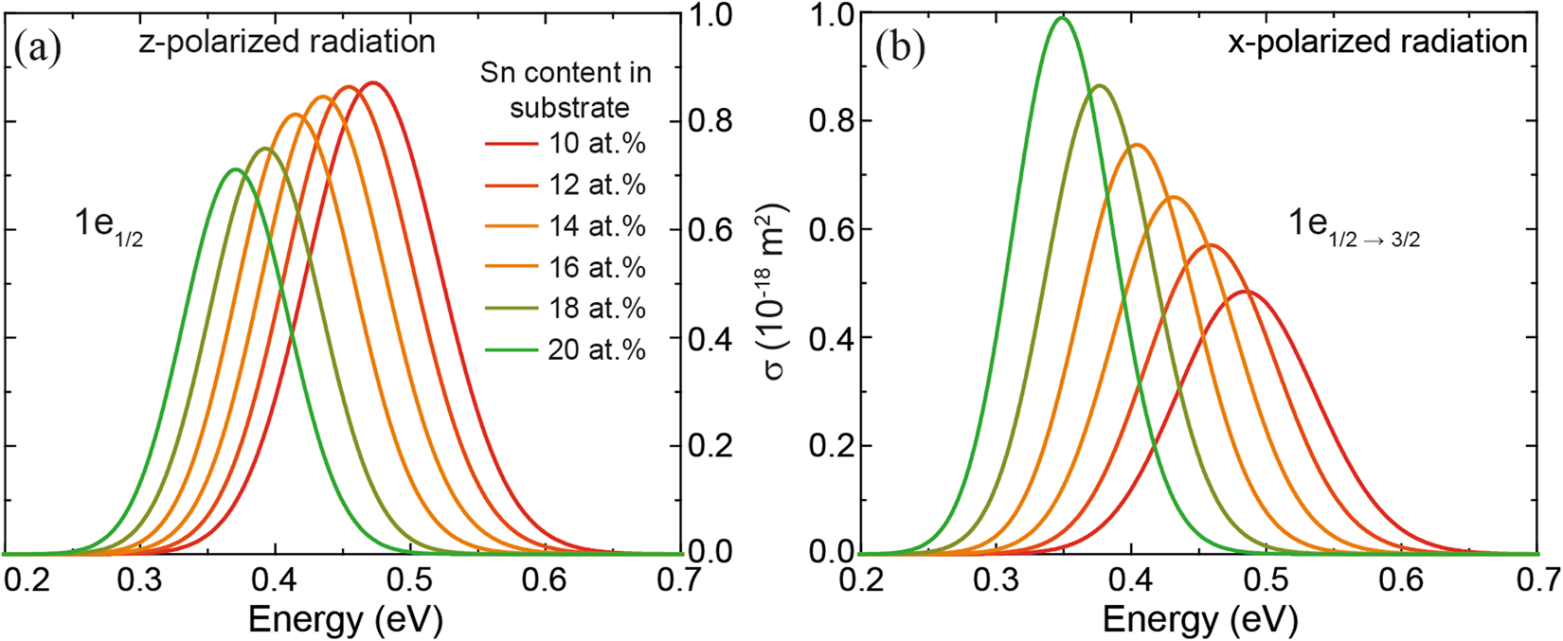
Optical cross section ${\sigma }_{if}^{\varepsilon }$ for z-polarized radiation for *Δm*_*f*_ = 0 (**a**) and *x-*polarized radiation for *Δm*_*f*_ = ±1 (**b**) in a Ge_0.82_Sn_0.18_/Si_0.10_Ge_0.74_Sn_0.16_ QD on various GeSn substrates.

**Figure 10:**
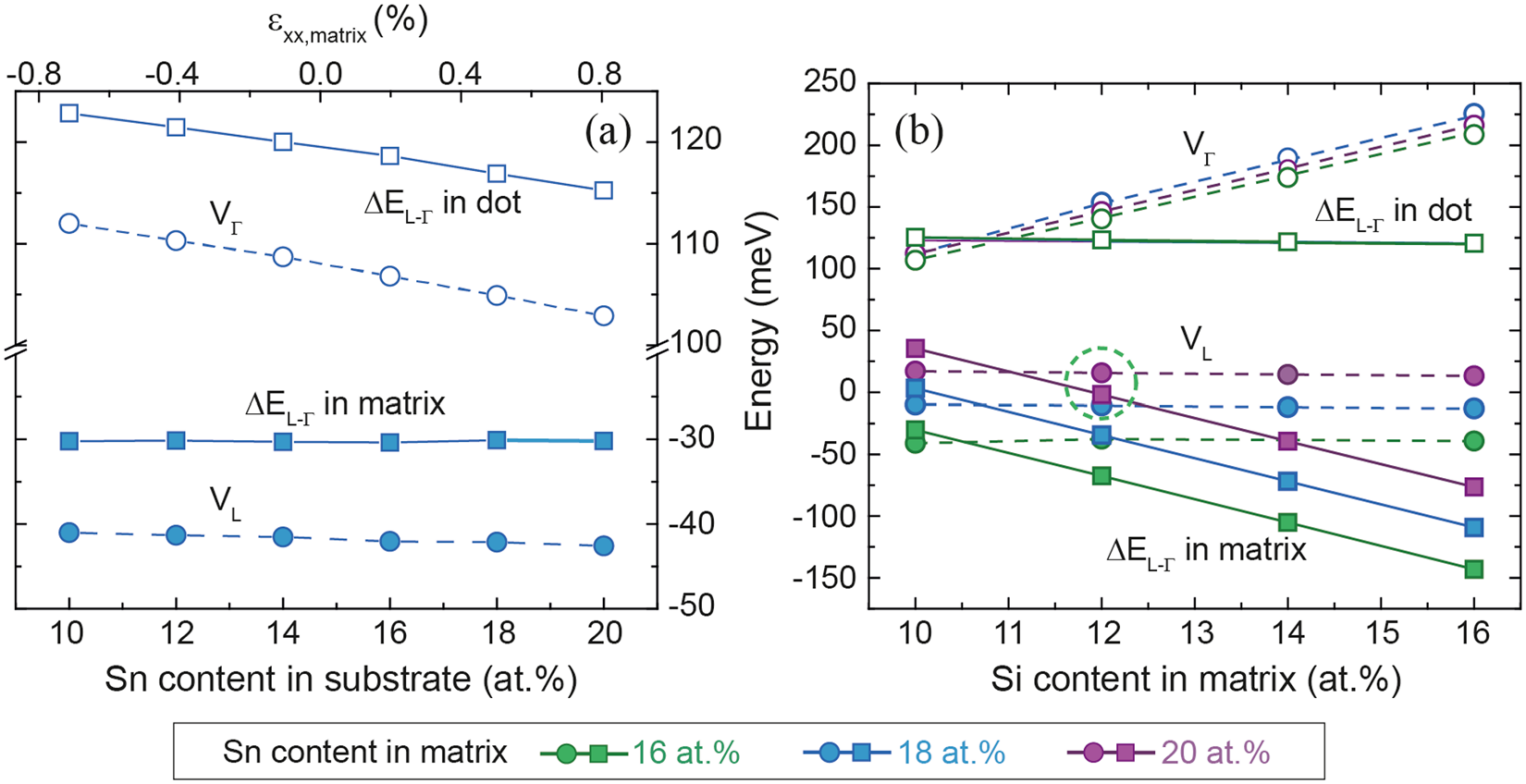
Influence of strain/Sn content in the substrate (**a**) and different Si/Sn contents in the SiGeSn matrix (**b**) on band discontinuities for Γ- (dashed lines, empty circles) and L-valley (dashed lines, solid circles) and directness *ΔE*_*L-Γ*_ in the matrix (solid lines, solid squares) and dot (solid lines, empty squares), in a Ge_0.82_Sn_0.18_/Si_0.10_Ge_0.74_Sn_0.16_ QD with 10 at.% in the substrate.

The optical cross section ${\sigma }_{if}^{\varepsilon }$ indicates how strongly two states are optically coupled. Since the focus of this work is the investigation of GeSn/SiGeSn heterostructures for light emitters, only transitions between the conduction and valence band were considered. The selection rule for the quasi angular momentum *m*_*f*_ for x-polarized light is *Δm*_*f*_ = ±1, while for *z-*polarized radiation it is *Δm*_*f*_ = 0. For the Ge_0.82_Sn_0.18_/Si_0.10_Ge_0.74_Sn_0.16_ QD it was found that for z-polarized radiation optical transitions between conduction band and valence band states are strongest for *|m*_*f*_*|* = 1/2. In the case of x-polarized light, transitions between *|m*_*f*_*|* = 1/2 and *|m*_*f*_*|* = 3/2 are strongest. In both cases transitions from the lowest conduction band state into the highest valence band state are the most important. In Fig. 9 we present the total optical cross section for emission of a carrier from the lowest state in the conduction band (having *|m*_*f*_*|* = 1/2) to states in the valence band. The results are shown for both polarizations and different Sn contents in the substrate. By increasing the Sn content in the substrate, the strain in matrix and dot turns from compressive into tensile, leading to lower band discontinuities and bandgaps (Fig. 10a). Moreover, the LH contribution of the highest valence band state decreases from 74% to 42%. Since for bulk material transitions from the conduction band into the LH band are allowed for z-polarized radiation, the fact that the LH contribution decreases implies that absorption of z-polarized radiation also decreases (Fig. 9a).

On the other hand, for x-polarized radiation the HH contribution of the highest valence band state increases from 53% to 82%, which implies that absorption of x-polarized radiation increases (Fig. 9b).

Applying biaxial compressive strain to the SiGeSn matrix chosen here unfortunately causes a type-II alignment between matrix and the GeSn dot for the L-valley and the valence bands. By increasing the Sn content in the substrate, tensile strain in the matrix causes a lower L-valley energy (Fig. 10a). This effect can be countered by increasing the Sn and adjusting Si content of SiGeSn, where the former shifts the strain towards compressive, increasing the L-valley energy, and the latter keeps the matrix indirect at the same time (Fig. 10b). The directness of the dot is not influenced by this change.

## Conclusion

This work presents the investigation of optimized material parameters for GeSn/SiGeSn heterostructures intended for use in efficient light emitting devices. It was shown, using 8-band k·p calculations, that sufficiently high carrier confinement can be achieved at 300 K for both GeSn/SiGeSn MQWs and GeSn QDs in a SiGeSn matrix. A broad range of epitaxially relevant Sn and Si contents were considered to determine ideal material configurations. In MQWs maximum band discontinuities and directnesses of 180 meV (~7 kT @ 300 K) and 160 meV, respectively, can be reached in wells with Sn contents of 16 at.%. For unstrained wells with Sn content above 16 at.%, the barrier becomes a direct bandgap semiconductor, which could lower radiative processes inside the well. Gain calculations for doped and intrinsic MQW structures indicate that considerable values of material net gain, around 480 cm^−1^, can be achieved despite FCA losses, for injection carrier densities exceeding 3 × 10^18^ cm^−3^ and doping levels of around 5 × 10^19^ cm^−3^.

For zero-dimensional quantum dot heterostructures, high band discontinuities above 105 meV for direct bandgap GeSn dots with a directness of 75 meV were found. Nevertheless, sufficient charge carrier confinement can be achieved in direct bandgap dots for (i) dots strained equally in every direction by the surrounding indirect bandgap matrix and (ii) in cases where the strain in the matrix is induced biaxially by a substrate.

## Methods

The k·p method calculates the band energies for a small range around the Γ point. It was introduced in ref.^35^ and extended to 8 bands (3 valence bands and 1 conduction band with different spin states) and the diamond/zinc blende lattice in refs^20,36^. In this work the formalism from ref.^20^ was used and the material parameters are given in Table 1.

**Figure 11:**
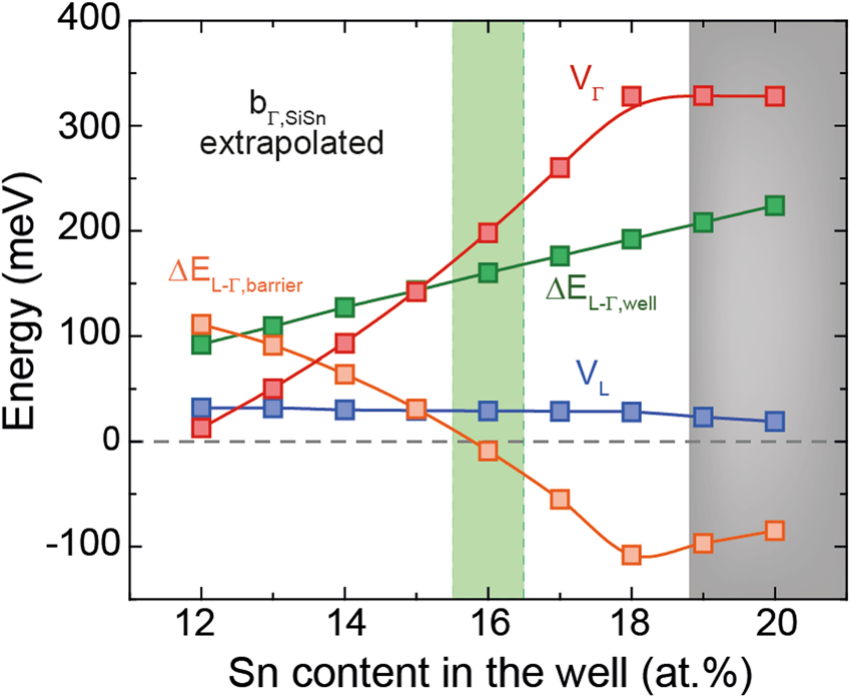
Recalculation of directness and highest band discontinuities of GeSn/SiGeSn heterostructures as presented in Fig. 3, but using the *b*_*Γ*,*SiSn*_ behavior extrapolated from ref.^37^.

Concerning band energy calculations, recent publications cast doubt on whether the SiSn bowing can be described by a single parameter, *b*_*Γ*,*SiSn*,_ because there is strong evidence that it depends on the Si and Sn concentration^37,38^. The origin of this behavior could be related to ordering effects in SiGeSn^39^. To compare our results with these experimental findings, we recalculated the carrier confinement for GeSn/SiGeSn MQWs using an extrapolation to higher Sn concentrations of the *b*_*Γ*,*SiSn*_ data presented in ref.^37^. For high Sn contents the bowing decreases to values in the range of the value we use for the calculations presented in this work (3.915 eV), so that the predictions in this region are effectively based on the latest experimental results. Applying the same procedure as in the main part of this work and shown in Fig. 3b, we get the results in Fig. 11. The main difference occurs for small Sn contents, where the band discontinuity at Γ is significantly smaller than for GeSn wells with high Sn contents. In the latter case the achievable band discontinuities are similar and even stronger than for *b*_*Γ*,*SiSn*_* = *3.915 eV. While the directness of the well is independent of *b*_*Γ*,*SiSn*_, the directness of the barrier decreases with increasing Sn content. The reason for this is that around 17 at.% Sn the bowing is smaller than 3.915 eV, shifting the directness of the SiGeSn barrier towards negative values. This has to be taken into account when finding the region of negative directness barriers. The extrapolation itself becomes critical at Sn contents exceeding ~19 at.% (grey area in Fig. 11), because it leads to negative bowings. It is doubtful if this is physically realistic. For the structure we have chosen for gain calculations (Fig. 4), the carrier confinement evaluation leads, for both bowing approaches, to similar results. Due to the lack of sufficient experimental data, in the presented calculations *b*_*Γ*,*SiSn*_ is chosen to be constant, which seems to be an adequate assumption, resulting in reasonable predictions.

In order to get band alignment of valence- and conduction bands in different layers, the Si/Sn content dependent average valence band energy was calculated for barriers and wells according to ref.^40^:

2 $${E}_{v,av}=-\,0.48\,eV\cdot {x}_{Si}+0.69\,eV\cdot {x}_{Sn}$$

In some recent publications different expressions from Eq. (2), ref.^40^, were used for *E*_*v*,*av*_^10,41^. Comparing the results from Fig. 3b with the corresponding results from these models shows no significant difference for the band alignment in the conduction band. Concerning the valence band, the average HH band discontinuity decreases from 104 to 63 meV, while for the LH band it increases from 40 to 50 meV. In any case, all three models lack a detailed experimental verification, and in all further calculations we have used Eq. (2) from ref.^40^. *E*_*v*,*av*_ and the split-off hole band energy *E*_*SO*_ is used to calculate the valence band top energy E_v_:

3 $${E}_{SO}=0.297\,eV\cdot {x}_{Ge}+0.044\,eV\cdot {x}_{Si}+0.8\,eV\cdot {x}_{Sn}$$

4 $${E}_{v}={E}_{v,av}+{E}_{SO}/3$$

and then, from Eq.(1), also the energies of valleys in the conduction band of a particular layer. Applying strain additionally shifts the valence- and conduction band energies by *ΔE*_*i*_, which can be calculated by:

5 $${\rm{\Delta }}{E}_{{\rm{\Gamma }}}={a}_{c}(2{\varepsilon }_{xx}+{\varepsilon }_{zz}),{\rm{\Delta }}{E}_{{\rm{L}}}={a}_{L}(2{\varepsilon }_{xx}+{\varepsilon }_{zz})$$

6 $${\rm{\Delta }}{E}_{{\rm{v}}}={a}_{v}(2{\varepsilon }_{xx}+{\varepsilon }_{zz}),{\rm{\Delta }}{E}_{{\rm{H}}{\rm{H}}}={b}_{v}({\varepsilon }_{xx}-{\varepsilon }_{zz})$$

7 $${\rm{\Delta }}{E}_{{\rm{LH}},{\rm{SO}}}=-\,{\rm{\Delta }}{E}_{{\rm{HH}}}-\frac{1}{2}({E}_{SO}+{\rm{\Delta }}{E}_{{\rm{HH}}}\mp \sqrt{{({E}_{SO}+{\rm{\Delta }}{E}_{{\rm{HH}}})}^{2}+{E}_{SO}+8{\rm{\Delta }}{{E}_{{\rm{HH}}}}^{2}})$$

where ε_xx_ and ε_zz_ can be calculated using the interpolated lattice constant *a*_*lattice*_ by:

8 $${\varepsilon }_{xx}=\frac{{a}_{lattice,substrate}-{a}_{lattice}}{{a}_{lattice}},{\varepsilon }_{zz}=-\,(2{C}_{12}/{C}_{11}){\varepsilon }_{xx}$$

9 $${a}_{lattice}={a}_{Ge}{x}_{Ge}+{a}_{Si}{x}_{Si}+{a}_{Sn}{x}_{Sn}+{b}_{SiGe}{x}_{Si}(1-{x}_{Si})+{b}_{GeSn}{x}_{Sn}(1-{x}_{Sn})$$

Since all these band energies, in different materials, have the same zero reference point (average valence band energy in unstrained Ge) the band discontinuities at interfaces are simply calculated as differences of the corresponding band/valley energies in the two materials.

Gain calculations for the Ge_0.84_Sn_0.16_/Si_0.10_Ge_0.75_Sn_0.15_ MQW structure discussed in the main part of this contribution were performed using Eq. 9.4.14 from ref.^42^, derived from k·p method:

10 $$\begin{array}{c}g(\hslash \omega )=\frac{\pi {e}^{2}}{{n}_{r}c{\varepsilon }_{0}{m}_{0}^{2}\omega }\sum _{n,\,m}{|{I}_{hm}^{en}|}^{2}{\int }_{0}^{\infty }{\rho }_{r}^{2D}{|\hat{e}\cdot {{\boldsymbol{p}}}_{cv}|}^{2}\frac{\gamma /\pi }{{[{E}_{hm}^{en}+{E}_{t}-\hslash \omega ]}^{2}+{\gamma }^{2}}\\ \,\,\,\,\,\times \,[{f}_{c}^{n}({E}_{t})-{f}_{v}^{m}({E}_{t})]d{E}_{t}\end{array}$$

where $|\hat{e}\cdot {{\boldsymbol{p}}}_{cv}|$ is the interband momentum matrix element, given in section 9.5.3 of ref.^42^, for transitions between c.b.-HH and c.b.-LH quantized states and 2*γ* is the (carriers scattering induced) broadening FWHM of the gain spectrum, chosen here to be 30 meV. For wells with thickness *L*_*z*_ the density of states ${\rho }_{r}^{2D}$ is defined using the reduced effective mass ${m}_{r}^{\ast }$ as:

11 $${\rho }_{r}^{2D}=\frac{{m}_{r}^{\ast }}{\pi {\hslash }^{2}{L}_{z}}$$

${I}_{hm}^{en}$ is the overlap integral of conduction- and valence-band envelope functions ${\varphi }_{n}$ and ${g}_{m}$ for electron and hole states ${e}_{n}$ and ${h}_{m}$:

12 $${I}_{hm}^{en}={\int }_{-\infty }^{\infty }{\varphi }_{n}(z){g}_{m}(z)dz$$

The Fermi-Dirac distributions ${f}_{c,v}^{n}$ for electrons and holes with the quasi-Fermi levels ${F}_{c,v}$ are defined as:

13 $${f}_{c}^{n}({E}_{t})=\frac{1}{1+exp[({E}_{g}+{E}_{en}+\frac{{m}_{r}^{\ast }}{{m}_{e}^{\ast }}{E}_{t}-{F}_{c})/{k}_{B}T]}$$

14 $${f}_{v}^{n}({E}_{t})=\frac{1}{1+exp[({E}_{g}+{E}_{hm}+\frac{{m}_{r}^{\ast }}{{m}_{h}^{\ast }}{E}_{t}-{F}_{v})/{k}_{B}T]}$$

For strain balanced MQWs with *n*_*well*_ wells and *n*_*barrier*_ barriers, the barrier thickness d_barrier_ can be expressed in terms of other parameters of the structure, as:

15 $${d}_{barrier}=-\frac{{n}_{well}}{{n}_{barrier}}\cdot \frac{{A}_{well}({x}_{Si},\,{x}_{Sn})}{{A}_{barrier}({x}_{Si},\,{x}_{Sn})}\cdot \frac{{a}_{barrier}({x}_{Si},\,{x}_{Sn})}{{a}_{well}({x}_{Si},\,{x}_{Sn})}\cdot \frac{{\varepsilon }_{well}}{{\varepsilon }_{barrier}}\cdot {d}_{well}$$

16 $${A}_{i}={C}_{11}^{(i)}+{C}_{12}^{(i)}-\frac{2{C}_{12}^{(i)2}}{{C}_{11}^{(i)}}$$

with ${C}_{11/12}^{(i)}$ being the elastic constants, *a*_*i*_ the unstrained lattice constants and *ε*_*i*_ the strain.

The optical cross section ${\sigma }_{if}^{\varepsilon }\,\,$used in this work for QD calculations is defined as:

17 $${\sigma }_{if}^{\varepsilon }(\omega )=\frac{2\pi }{\bar{n}c{\varepsilon }_{0}\omega }{|{M}_{if}^{\varepsilon }|}^{2}\frac{1}{\sigma \sqrt{2\pi }}\exp (\frac{{({E}_{f}-{E}_{i}-\hslash \omega )}^{2}}{2{\sigma }^{2}})$$

with the angular frequency $\omega$, the refractive index $\bar{n}$, the speed of light in vacuum *c*, the vacuum dielectric permittivity ${\varepsilon }_{0}$, the initial and final state energies ${E}_{i}$ and ${E}_{f}$, the polarization dependent transition matrix element ${M}_{if}^{\varepsilon }$ and the broadening factor $\sigma$. Since there are no reliable data on the peak broadening for GeSn quantum structures, the broadening factor $\sigma$ was chosen here to be 10% of the transition energy, same as used for III-V semiconductor QDs^22^.

The datasets generated during and/or analysed during the current study are available from the corresponding author on reasonable request.
